# The *KMT2A/MLL* consensus gene structure: a comprehensive update for research and diagnostic implications

**DOI:** 10.1038/s41375-024-02261-3

**Published:** 2024-04-27

**Authors:** C. Meyer, P. Larghero, B. A. Lopes, R. Marschalek

**Affiliations:** 1 Goethe-University, Institute of Pharm. Biology/DCAL, Frankfurt/Main, Germany; 2 GenLAb/Program of Molecular Carcinogenesis, Instituto Nacional de Câncer (INCA), Rio de Janeiro, RJ Brazil

**Keywords:** Genetics research, Haematological cancer

## To the Editor:

The *KMT2A* gene holds significant importance in both basic research and clinical diagnosis due to its involvement in numerous chromosomal translocations. To date, a total of 112 such translocations have been identified at the molecular level, all linked with the onset of acute leukemias presenting different disease phenotypes (ALL, AML, MPAL) [1].

The *KMT2A* gene, initially identified as the *MLL* gene located at chromosomal position 11q23 [2], was subsequently characterized by several other laboratories in 1992 and 1993. Due to its homology with the Drosophila Trithorax protein, it was designated as the “Trithorax-like gene” [3], “ALL-1” [4], and the human homolog of Drosophila Trithorax, “HRX” [5].

In 1996, two independent labs elucidated the complete gene structure of *KMT2A*. Using cloned radiolabeled cDNAs, Carlo Croce’s and our laboratory generated slightly differing provisional gene structures [6, 7]. Notably, the presence or absence of *KMT2A* exon 2 varies in public databases (NCBI: NG_027813; ENSEMBL: ENSG00000118058). However, a quantitative RNA-Seq data analysis demonstrated that *KMT2A* exon 2, spanning only 99 bp, is as frequently observed in RNA-Seq data as neighboring *KMT2A* exons 1 (431 bp) and exon 3 (70 bp), indicating that it is rarely spliced out. ENSEMBL lists exon 2 only in two full-length transcripts of KMT2A (ENST00000531904 and ENST00000649666).

In addition to the discrepancies concerning *KMT2A* exon 2, a recent experimental analysis unveiled a previously unidentified “exon 22” situated within intron 21 of *KMT2A*. This newly discovered *KMT2A* exon 22 has been documented in the ENSEMBL database (ENST00000691053), indicating its potential incorporation into rare and alternatively spliced transcripts.

Since public databases do not yet faithfully represent the correct *KMT2A* gene structure, we advocate for an updated *KMT2A* gene structure comprising a total of 38 exons for the scientific community. Additionally, we provide precise nucleotide positions of all *KMT2A* exons and introns on chromosome 11, based on the GRCh38/hg38 human reference genome, in a Supplementary Data File.

## Description of the *KMT2A* gene and its alternative splice products

**Fig. 1:**
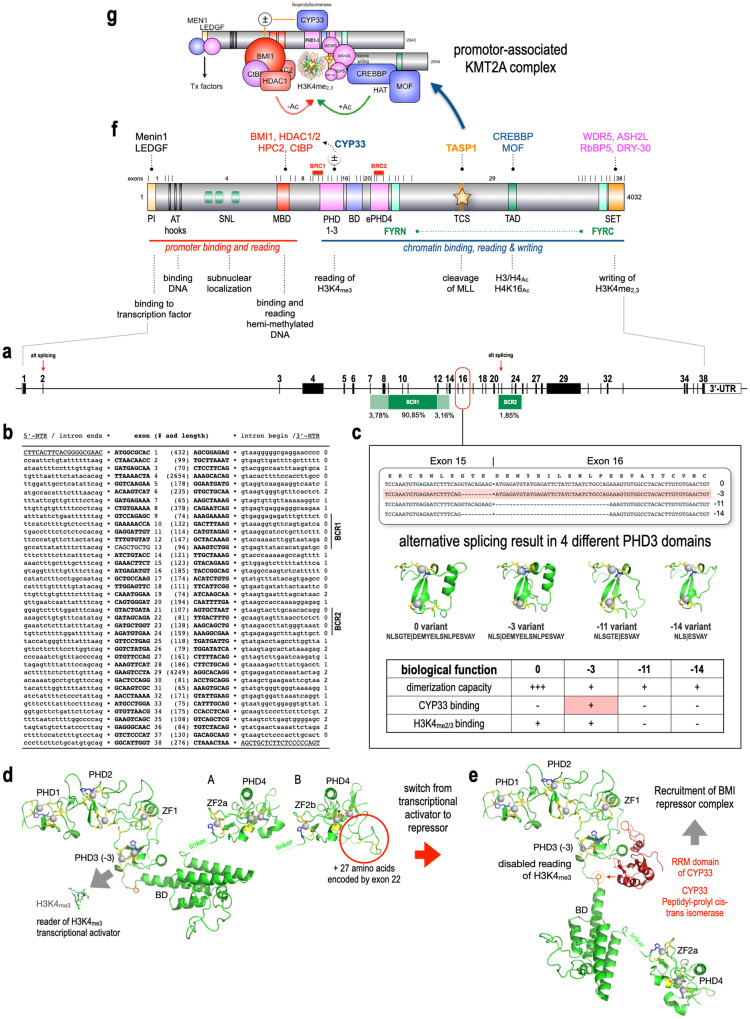
The KMT2A gene structure, protein domain structure and important alternative splice variants. **a** *KMT2A* gene structure with percentages of breakpoints in BCR1 and BCR2. **b** the 5‘-UTR/intron • exon and exon • intron/3’-UTR junctions of all 38 exons. The gene has a maximal open reading frame of 12,096 nucleotides (4032 amino acids), but exhibits also alternative protein variants due to alternative splicing events that may exclude exon 2 or 22, as well as the junction between exon 15 and 16 (4005 aa minus 3/11/14 or 3972 aa minus 3/11/14. **c** summarizes the alternative splice events at the exon 15/16 junction, leading to functional diverse KMT2A PHD3 domains. The depicted protein structures have been folded in silico by using the SWISS-Model server. **d** The PHD1-3/BD/extPHD4 domain of KMT2A. Predicted structural folding of the PHD1-3/BD/extPHD4 domains with depicted either the −3 variant of PHD3 (A) or with the additional 27 amino acids encoded by the alternatively spliced exon 22 (B). This changes ZF2a into ZF2b and displays an extended folding below the PHD4 domain structure which remains structurally unchanged, but contains two cysteines. **e** Structure of the CYP33 bound region, where the position of the BD/extPHD4 domain is changed due to a cis-trans isomerization of a proline residue located between the PHD3 and BD domain. This disables H3K4_me3_ reading, but allows CYP33 binding and the recruitment of the BMI1 repressor complex to the CXXC domain of KMT2A. All depicted protein domain structures have been folded in silico by using the SWISS-Model server. **f** KMT2A protein. All domains of the KMT2A protein are depicted. Above the protein structure of 4032 amino acids: exon boundaries for all 38 exons and the known protein binding partners. Below the protein structure: known molecular functions of these protein modules. **g** Taspase1 cleavage at the 2 Taspase1 cleavages sites (TCS) converts the full-length protein into an N- and C-terminal portion that form a complex with all protein binding partners. The final complex is binding and reading promoters, as well as able to bind, read and write chromatin signatures. If the Polycomb repressor complex (BMI1, HPC2, CtBP and HDAC1) is recruited via the CYP33-induced conformational switch in PHD3, then this protein complex converts from a transcriptional activator to a transcriptional repressor complex.

As illustrated in Fig. 1a, the *KMT2A* gene comprises 38 exons, including the 5’- and 3’-unstranslated regions (UTR), distributed across a 90,375 bp region at 11q23.3. The core of the major breakpoint cluster region (BCR1) lies between *KMT2A* exons 9 and 12 (90,85%), flanked by fewer breakpoints located at adjacent up- or downstream exons and introns (6,94%), while breakpoints in the minor BCR2 are mostly located between *KMT2A* exons 21–25 (1,85%). Breakpoints outside these areas are rare but do exist (0,42%). Figure 1a highlights also the 2 additional exons, which are both absent in the Mane (Matched Annotation from NCBI and EMBL-EBI) transcripts provided by public databases. In Fig. 1b, the 5’UTR, intron•exon and exon•intron juctions, as well as the 3’-UTR extremities of all 38 *KMT2A* exons are depicted by short sequence stretches, aiding in the identification of corresponding exons during sequence analysis or comparison to public databases. Based on these data, alternative splicing rarely occurs for *KMT2A* exon 2, but frequently for *KMT2A* exon 22.

Notably, an important biological function arises from alternative splice events at the *KMT2A* exon 15/16 junction [8]. These events yield four distinct protein products: the 0, −3, −11, and −14 variants. Utilizing SWISS-MODEL [9], the four different PHD3 domain variants were analyzed for their folded protein structure (Fig. 1c). These variants induce subtle changes in the KMT2A PHD3 domain’s molecular properties and its interaction with the adjacent bromodomain. The KMT2A PHD1–3 domain has been described to confer oligomerization [10], and to bind to the CYP33 protein (a peptidyl-prolyl cis-trans isomerase) [11]. In addition, it is a “reader domain” for H3K4_me2/3_ [12] which is greatly enhanced by the adjacent Bromodomain [13]. A tiny linker of only 8 amino acids (N-TERHPAEW-C) separates both domains and contains the important proline residue that allows to change the interaction of both domains.

The folded 0- and −3-variants display an alpha-helix crucial for docking to the BD domain, while the −11- and −14-variants lack this structural element, suggesting an alternative conformation incompatible with BD domain docking. The tiny loop structure next to the alpha helix in the 0-variant appears to block CYP33 binding, emphasizing its role in binding and reading solely H3K4_me3_ signatures. However, the 0-variant demonstrates the strongest dimerization capacity among variants in yeast-2-hybrid experiments [8]. The −3-variant exhibits an alpha-helix but lacks the tiny loop structure, yet it binds to H3K4_me3_ signatures in the absence of CYP33. Additionally, it displays weak binding activity to other variants in Y-2-H experiments. The −11 and −14-variants exhibit weaker dimerization capacity due to distorted or missing alpha-helices.

SWISS-MODEL was also used to model the complete PHD/BD domain (Fig. 1d, A). Incorporating the new exon 22 coding sequences resulted in a slightly different structure prediction in the extended PHD4 domain (Fig. 1d, B), disrupting a small alpha-helix and substituting it with a distorted amino acid stretch containing two cysteines.

CYP33 binding to PHD3 induces a dramatic structural change, catalyzing a cis-to-trans conversion of the Pro-1665 residue in the linker region between PHD3 and the adjacent Bromodomain (Fig. 1d). This refolded PHD3/Bromodomain loses its ability to bind to H3K4_me2/3_ signatures [13] and recruits a Polycomb repressor complex to the CXXC domain of KMT2A. Thus, binding of BMI1 repressor complex transforms the KMT2A protein complex from a transcriptional repressor, thereby preventing binding of MLL to target genes like *HOXA9* and *HOXC8*.

## Description of the KMT2A protein and its protein interactions

The KMT2A protein, depicted in Fig. 1f, comprises distinct domains with specific functions in gene transcription and chromatin activation. These functions are crucial for regulating target genes during development and maintaining tissue-specific gene expression in differentiated cells. The KMT2A protein consists of the N-terminal portion responsible for promoter binding and reading, and the C-terminal portion involved in chromatin binding, reading, and writing, separated by the red-marked breakpoint cluster region 1 (BCR1).

Notably, cleavage of KMT2A protein by Taspase1 at two cleavage sites (TCS) results in two protein fragments: MLL-N (with a molecular weight of 320 kDa) and MLL-C (with a molecular weight of 180 kDa). These fragments form a stable complex via the FYRN and FYRC domains, contributing to the functional KMT2A complex depicted in Fig. 1g, either with or without the BMI1 repressor complex. Chromosomal breakpoints within BCR1 disrupt the KMT2A protein, allowing the N-terminal portion to bind to target gene promoters via the MEN1/LEDGF complex at the very N-terminus. Conversely, the reciprocal portion retains chromatin binding, reading, and writing capabilities but in a manner independent of promoter mediation. Reciprocal fusion proteins, such as AFF1::KMT2A and AFDN::KMT2A exhibit potent chromatin opening functions, enabling the direct fusion proteins KMT2A::AFF1 and KMT2A::AFDN to activate approximately 12-times more target genes [14, 15].

Chromosomal breakpoints in BCR2 of the *KMT2A* gene likely disrupt the PHD4 domain.

## Explaining the Supplementary Data File

This paper provides additional data and information in a separate Excel file. This document includes details about the *KMT2A* gene and all yet known partner genes of direct *KMT2A* fusion alleles (*n* = 112) or *KMT2A*::PTDs. The individual 115 sheets comprise the *KMT2A* gene (sheet 1), while sheet 2 is listing the frequency of fusion partners (diagnosed at the DCAL and literature). All other sheets contain information about the 112 known fusion partner genes (sheets 3–114) or *KMT2A*::PTDs (sheet 115).

Each page begins with the gene name, its known biological function, known alias names, chromosomal location, and the number of exons based on common gene structures within public databases, the described leukemia disease phenotypes and the number of identified breakpoints at the DCAL and literature. Following this, a graphic of the respective gene structure (left to the right) displaying a heatmap of chromosomal breakpoints on top. They are either indicated in blue (if only the intron has been mapped in the literature) or in red (if the precise chromosomal breakpoint has been sequenced).

Subsequently, a table is provided for each gene defining all structural elements (exons and introns), including the 5’- and 3’-UTR. These tables provide the cytoband, gene symbol, precise nucleotide information of all structural elements, transcribed strand, and exon phase (0, 1, or 2), indicating how the reading frame is disrupted as consequence of a gene fusion. Finally, all identified breakpoints for each structural element are shown in numbers (n) and percentages (Freq). Additionally, the gene length and the number of identified breakpoints are provided at the bottom of the table.

Further information of all patients added from the literature is available in a second table on the right side. This additional table provides information of respective patients regarding PMID, Intron/Exon, disease phenotype, gender and age. If either the total number of patients is ≤50 or the breakpoint was located outside the major BCR1 this information is also given for DCAL samples.

Therefore, we provided novel data which is beneficial for all centers seeking to automate their routine diagnostic units, particularly for identifying and analyzing fusion mRNAs of the *KMT2A* gene in RNA-Seq data of their patients. This will contribute to advancing science and diagnosis, aiding in the design of appropriate molecular probes to improve outcomes for leukemia patients.

### Supplementary information

Supplementary File

## Data Availability

More detailed breakpoint information and data from the investigated acute leukemia patients can be made available to scientist upon request.
